# Simultaneous Degradation of AFB1 and ZEN by CotA Laccase from *Bacillus subtilis* ZJ-2019-1 in the Mediator-Assisted or Immobilization System

**DOI:** 10.3390/toxins16100445

**Published:** 2024-10-16

**Authors:** Boquan Gao, Wei An, Jianwen Wu, Xiumin Wang, Bing Han, Hui Tao, Jie Liu, Zhenlong Wang, Jinquan Wang

**Affiliations:** 1 Key Laboratory of Feed Biotechnology, Ministry of Agriculture and Rural Affairs, Institute of Feed Research, Chinese Academy of Agricultural Sciences, No. 12 Zhongguancun South Street, Beijing 100081, China; 2 Laboratory of Pet Nutrition and Food, Institute of Feed Research, Chinese Academy of Agricultural Sciences, No. 12 Zhongguancun South Street, Beijing 100081, China

**Keywords:** CotA, AFB1, ZEN, degradation, immobilized

## Abstract

The global prevalence of aflatoxin B1 (AFB1) and zearalenone (ZEN) contamination in food and feed poses a serious health risk to humans and animals. Recently, enzymatic detoxification has received increasing attention, yet most enzymes are limited to degrading only one type of mycotoxin, and free enzymes often exhibit reduced stability and activity, limiting their practicality in real-world applications. In this study, the laccase *CotA* gene from ZEN/AFB1-degrading *Bacillus subtilis* ZJ-2019-1 was cloned and successfully expressed in *Escherichia coli* BL21, achieving a protein yield of 7.0 mg/g. The recombinant CotA (rCotA) completely degraded AFB1 and ZEN, with optimal activity at 70 °C and pH 7.0. After rCotA treatment, neither AFB1 nor ZEN showed significantly cytotoxicity to mouse macrophage cell lines. Additionally, the AFB1/ZEN degradation efficiency of rCotA was significantly enhanced by five natural redox mediators: acetosyringone, syringaldehyde, vanillin, matrine, and sophoridin. Among them, the *acetosyringone*-rCotA was the most effective mediator system, which could completely degrade 10 μg of AFB1 and ZEN within 1 h. Furthermore, the chitosan-immobilized rCotA system exhibited higher degradation activity than free rCotA. The immobilized rCotA degraded 27.95% of ZEN and 41.37% of AFB1 in contaminated maize meal within 12 h, and it still maintained more than 40% activity after 12 reuse cycles. These results suggest that media-assisted or immobilized enzyme systems not only boost degradation efficiency but also demonstrate remarkable reusability, offering promising strategies to enhance the degradation efficiency of rCotA for mycotoxin detoxification.

## 1. Introduction

Mycotoxins, mainly including aflatoxin B1(AFB1), zearalenone (ZEN), deoxynivalenol (DON), ochratoxin A and fumonisins, are secondary metabolites primarily produced by various fungi such as *Penicillium*, *Aspergillus*, and *Fusarium* [1]. They are prevalent in crops, feed, and food worldwide [2], posing a serious threat to human and animal health and causing huge economic losses to the food and livestock industries [3,4]. AFB1, recognized for its potent hepatotoxic and mutagenic effects, can cause severe liver damage and has been classified as a Group A carcinogen by the International Agency for Research on Cancer (IARC) [5,6]. ZEN is a non-steroidal female mycotoxin with strong reproductive, immunotoxic, hepatotoxic, and genotoxic effects. After ingestion, ZEN and its metabolites can competitively bind to estrogen receptors and cause estrogen syndrome symptoms in animals, leading to infertility, abortion, and stillbirth [7]. ZEN is also a potent carcinogen, posing a serious threat to animal and human health [8].

Although the threat of AFB1 and ZEN to livestock health can be mitigated to some extent by dietary control, their presence in livestock cannot be avoided [9]. Various physical and chemical detoxification approaches have been proposed to control AFB1/ZEN contamination [10,11]. However, completely eliminating AFB1 and ZEN is challenging due to their inherent stability under UV light, temperature variations, and acidic conditions. Moreover, residual chemicals not only destroy the nutrients of feed and seriously affect the taste of food, but also produce secondary toxic compounds and pollute the environment [12]. Most conventional adsorbents with effective adsorption capacity for AFB1 show weak adsorption capacity for ZEN and other fungal toxins, thereby restricting their utility for broad-spectrum detoxification [13]. There is an urgent need to develop new effective strategies to reduce mycotoxin contamination. In recent years, researchers have paid more attention to biological strategies, including the use of microorganisms and enzymes.

Recent studies have shown that several enzymes can effectively degrade ZEN and AFB1. For example, Liu et al. identified an *Armillariella tabescens* multienzyme from edible mushrooms, which is responsible for opening the difuran ring of AFB1 and minimized the toxicity of AFB1 [14]. Taylor et al. discovered an F420H2-dependent aflatoxin reductase from *Mycobacterium smegmatis*, an enzyme that selectively targets the α/β-unsaturated ester moiety of AFB1 [15]. Additionally, some ZEN-degrading enzymes have been identified from bacterial strains, such as *Clonostachys rosea* (lactonase: ZHD101), *Acinetobacter Sp SM04* (peroxidases: Prx), and *Bacillus subtilis* (laccases: BsCotA) [16,17,18]. The lactonase ZHD101 has been extensively studied for its high ZEN degradation capacity, but poor thermal stability limits its further application [19,20].

The laccase is a class of oxidoreductases with a mononuclear copper center and a TNC (type 1 copper site) that was first discovered in 1883 by Hikorokuro Yoshida from *Rhus vernicifera* [21] and has attracted more attention due to its high efficiency in degrading AFB1 and ZEN simultaneously and its environmentally friendly properties [22,23,24]. Guo et al. reported that *B. licheniformis* ANSB821 CotA laccase could catalyze the direct oxidation of AFB1 and ZEN with 65%–96% degradation rate [25]. In addition, most of the plant extracts were confirmed to assist degradation of AFB1 and ZEN by CotA. For example, methyl syringate was an efficient mediator assisting *Bacillus subtilis* CotA laccase to degrade AFB1 (98.0%) and ZEN (100.0%) [26]. *Pleurotus eryngii* Ery4 laccase was also reported to degrade AFB1 and ZEN in the presence of redox mediators [27]. However, whether the mediator system is a universal approach to enhance laccase activity, especially in mycotoxin degradation, still needs to be investigated on different sources of laccase.

Although laccase reported in recent years have shown efficient mycotoxin rates, the application in mycotoxin detoxification remains challenging [23]. This can be attributed to some of the limitations of enzyme-mediated catalysis, particularly their sensitivity to environmental conditions such as pH and temperature and intolerance to organic solvents [28,29]. To cope with these limits, enzyme immobilization techniques are commonly employed, where the enzyme is cross-linked, attached to a support, embedded in a polymer network, encapsulated or chemically modified [30]. For instance, Tomás et al. used muNS-Mi nanospheres to immobilize CotA laccase, resulting in an extended pH working range and the ability to recycle without activity loss [31]. Similarly, Bo et al. constructed a biomimetic core–shell PDA@Lac by immobilizing CotA laccase with polydopamine encapsulation, and the relative activity of PDA@Lac maintained approximately 75% after 10 reuse cycles [32].

In the previous study, *Bacillus subtilis* strain ZJ-2019-1 was found to be effective in degrading AFB1 and ZEN [33]. In this work, the laccase CotA gene from *Bacillus subtilis* ZJ-2019-1 was heterologously expressed in *E. coli* BL21. We systematically investigated various factors affecting the activity of recombinant CotA against AFB1 and ZEN, including culture conditions, temperature, pH, and enzyme concentration. In addition, we investigated the ability of recombinant CotA to degrade AFB1 and ZEN in mediator-assisted or immobilization systems and explored the preliminary application of immobilized-CotA for detoxification in contaminated maize meal.

## 2. Results

### 2.1. Cloning, Expression, and Purification of Laccase CotA from Bacillus subtilis ZJ-2019-1

**Figure 1:**
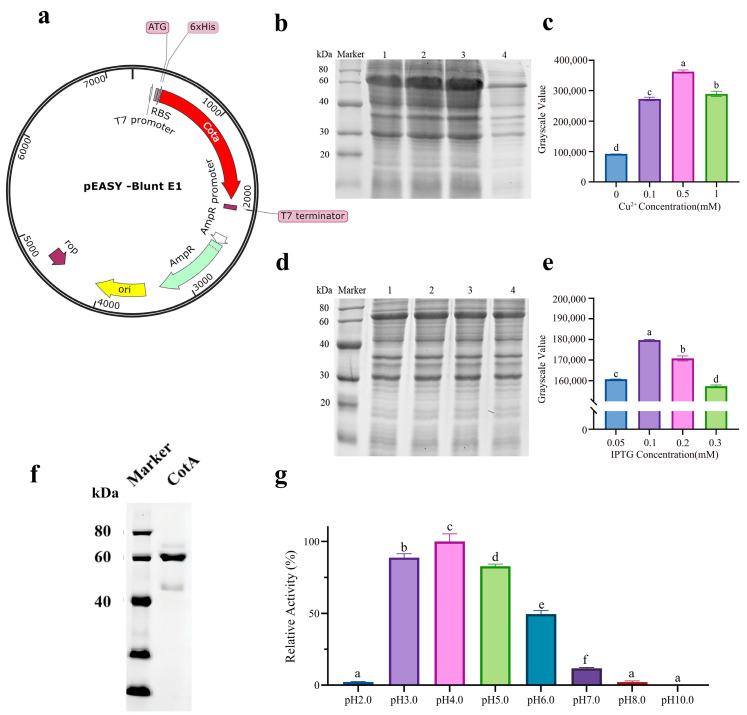
Expression optimization, purification, and characterization of rCotA: (**a**) Construction of the pEASY-Blunt E1-cotA plasmid. (**b**) SDS-PAGE analysis showing rCotA expression with varying IPTG concentrations. Lane 1: 0.05 mM IPTG; Lane 2: 0.1 mM IPTG; Lane 3: 0.2 mM IPTG; Lane 4: 0.3 mM IPTG. (**c**) Densitometric analysis of the SDS-PAGE gel bands for IPTG induction. Different lower-case letters indicate a significant difference between the two groups (*p* < 0.05). (**d**) SDS-PAGE analysis of rCotA expression with different Cu^2+^ concentrations. Lane 1: 0.1 mM Cu^2+^; Lane 2: 0.5 mM Cu^2+^; Lane 3: 1.0 mM Cu^2+^; Lane 4: 0 mM Cu^2+^. (**e**) Densitometric analysis of SDS-PAGE gels for Cu^2+^ induction. Different lower-case letters indicate a significant difference between the two groups (*p* < 0.05). (**f**) SDS-PAGE analysis of purified rCotA showing a single band at 60 kDa. (**g**) Enzymatic activity of purified rCotA at different pH levels (2.0–10.0) using ABTS as a substrate. Different lower-case letters indicate a significant difference between the two groups (*p* < 0.05).

The open reading frame length of the *Bacillus subtilis* ZJ-2019-1 laccase CotA gene was 1542 bp, encoding 513 amino acids with a predicted molecular weight of 59.1 kDa and an isoelectric point of 6.25. The recombinant plasmid *pEASY*-Blunt E1-CotA was successively transformed into *E. coli* BL21 (DE3) cells for expression and purified by Ni-NTA column (Figure 1a). We optimized the expression conditions of rCotA and investigated the effects of different concentrations of IPTG and Cu^2+^ on its expression. The optimal IPTG concentration for inducing expression was 0.1 mM (Figure 1b,c), and the optimal Cu^2+^ concentration was 0.5 mM (Figure 1d,e), as determined by gel analysis. The purified rCotA showed a clear single band with an apparent molecular weight of 60 kDa (Figure 1f), which is consistent with the predicted size of the N-terminal 6 × His-tagged CotA protein, indicating successful expression and purification of *Bacillus subtilis* ZJ-2019-1CotA in *E. coli*. In addition, using ABTS as a substrate, the enzyme activity of purified rCotA was 247.2 U/L at pH = 4 (Figure 1g).

### 2.2. The Degradation Characteristics of rCotA on AFB1 and ZEN

**Figure 2:**
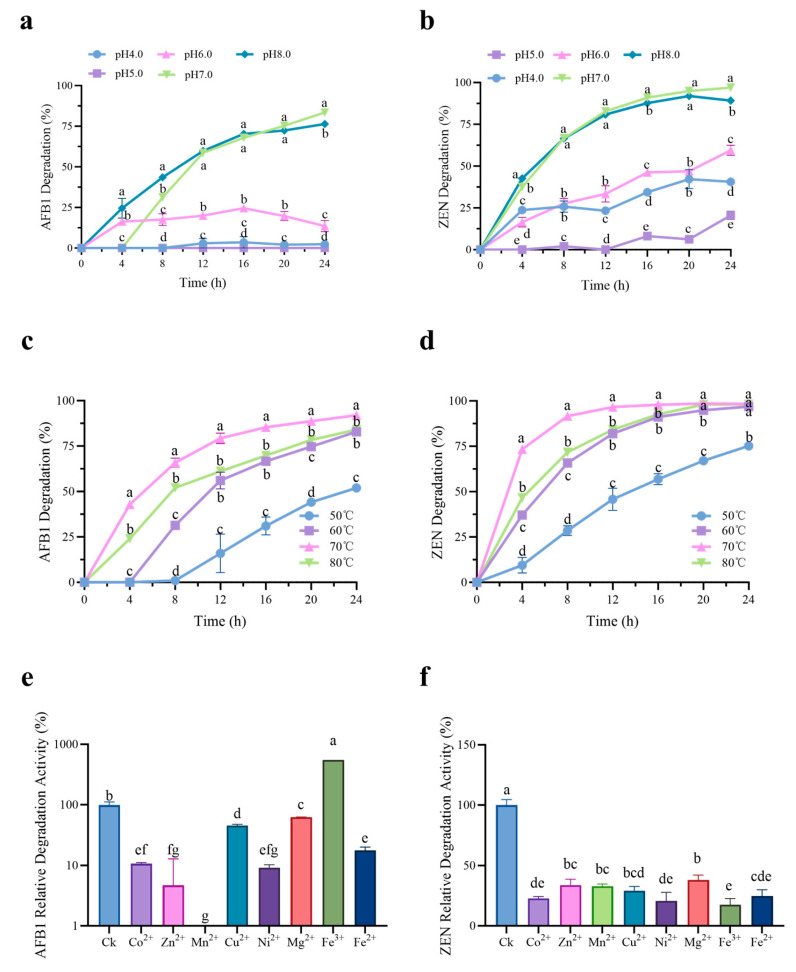
Degradation efficiency of AFB1 and ZEN by rCotA under varying conditions. (**a**) Time-course analysis of AFB1 degradation by rCotA at different pH. (**b**) Time-course analysis of ZEN degradation by rCotA at different pH. (**c**) Time-course analysis of AFB1 degradation by rCotA at different temperatures. (**d**) Time-course analysis of ZEN degradation by rCotA at different temperatures. (**e**) Time-course analysis of AFB1 degradation by rCotA with different metal ions. (**f**) Time-course analysis of ZEN degradation by rCotA with different metal ions. The results are presented as the mean ± SD of three independent experiments. Different lower-case letters indicate a significant difference between the two groups (*p* < 0.05).

Time courses of AFB1 and ZEN degradation by purified rCotA under different pH and temperature were shown in Figure 2. The degradation rates of AFB1 were 83.43% (pH 7.0) and 76.41% (pH 8.0) at 24 h, respectively, significantly higher than those at pH 4.0–6.0 with 0%–16.99% (Figure 2a). Similarly, rCotA showed a stronger ability to degrade ZEN at pH 7.0 (96.95%) and pH 8.0 (89.24%) at 24 h (Figure 2b). However, rCotA showed only weak to moderate ZEN degradation ability at pH 4.0 (40.64%), pH 5.0 (20.52%), and pH 6.0 (59.44%) at 24 h. It is worth noting that rCotA exhibited a higher capacity for degrading ZEN than AFB1 (Figure 2a,b). In addition, the effect of temperature on rCotA activity was also evaluated. Within a certain temperature range, the degradation rate of both AFB1 and ZEN significantly increased with rising temperature. The degradation rate of AFB1 was 8.07%–52.96% (50 °C), 50.85%–83.65% (60 °C), 77.33%–93.18% (70 °C), and 60.65%–84.40% (80 °C) over 12–24 h, respectively. Similarly, the degradation rate of ZEN was 40.54%–76.66% (50 °C), 80.19%–97% (60 °C), 96.32%–98.5% (70 °C), and 82.39%–98.29% (80 °C), respectively, over 12–24 h (Figure 2c,d). These results suggest that rCotA has a stronger ability to degrade AFB1 and ZEN at pH 7.0 and 70 °C. The effect of various ions on rCotA activity was further explored. While most ions significantly inhibited the AFB1/ZEN degradation activity of rCotA, Fe^3+^ notably enhanced AFB1 degradation by 5.5-fold. In contrast, Mn^2+^ completely inhibited the AFB1 degradation activity of rCotA. 

### 2.3. Degradation of AFB1 and ZEN by rCotA Mediator System

**Figure 3:**
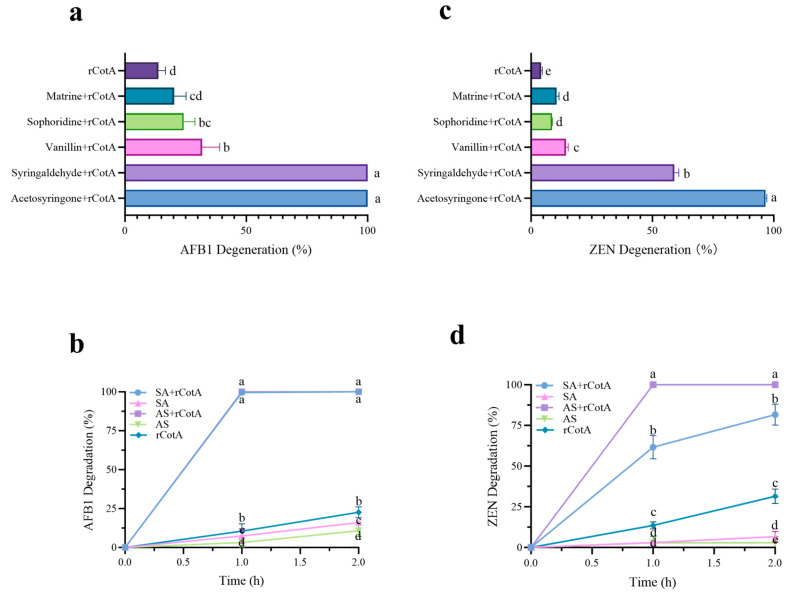
The effect of various mediators on the degradation of AFB1 and ZEN by rCotA. (**a**) AFB1 degradation rates in different rCotA mediator systems. (**b**) ZEN degradation rates in different rCotA mediator systems. (**c**) Time-course analysis of AFB1 degradation rates in the acetosyringone– and syringaldehyde–rCotA mediator system. (**d**) Time-course analysis of ZEN degradation rates in the acetosyringone– and syringaldehyde–rCotA mediator system. The results are presented as the mean ± SD of three independent experiments. Different lower-case letters indicate a significant difference between the two groups (*p* < 0.05).

Most mediators have been found to improve laccase activity and significantly increase the degradation efficiency of AFB1 and ZEN. In this study, five natural mediators (syringaldehyde, acetosyringone, vanillin, matrine, and sophoridin) were selected to investigate their impact on rCotA-mediated degradation. As shown in Figure 3a, the degradation rates of AFB1 and ZEN in the control group (rCotA without mediators) were 13.73% and 4.3%, respectively. In contrast, the presence of mediators significantly boosted the degradation of AFB1, with syringaldehyde and acetosyringone being the most effective, achieving 100% degradation. This was followed by vanillin (31.84%), sophoridin (24.39%), and matrine (20.23%). Similarly, ZEN degradation rates were markedly improved in the rCotA mediator system, reaching 96.6% with acetosyringone, 59% with syringaldehyde, 14.35% with vanillin, 10.51% with matrine, and 8.77% with sophoridin (Figure 3b). The time-course analysis of degradation was also investigated. In the absence of rCotA, acetosyringone and syringaldehyde monomers showed limited degradation efficiencies of 10.77%–16.03% for AFB1 (Figure 3c) and 2.92%–5.4% for ZEN (Figure 3d), respectively, after 2 h. In contrast, the acetosyringone–rCotA mediator system completely degraded 10 μg of AFB1 and ZEN within 1 h. For the syringaldehyde–rCotA system, AFB1 was completely degraded within 1 h, while ZEN degradation reached 58.29% at 1 h and 79% at 2 h. The results suggested that acetosyringone is the most effective mediator for simultaneous degradation of AFB1 and ZEN. 

### 2.4. Toxicity Analysis of rCotA-Mediated AFB1 and ZEN Degradation Products

**Figure 4:**
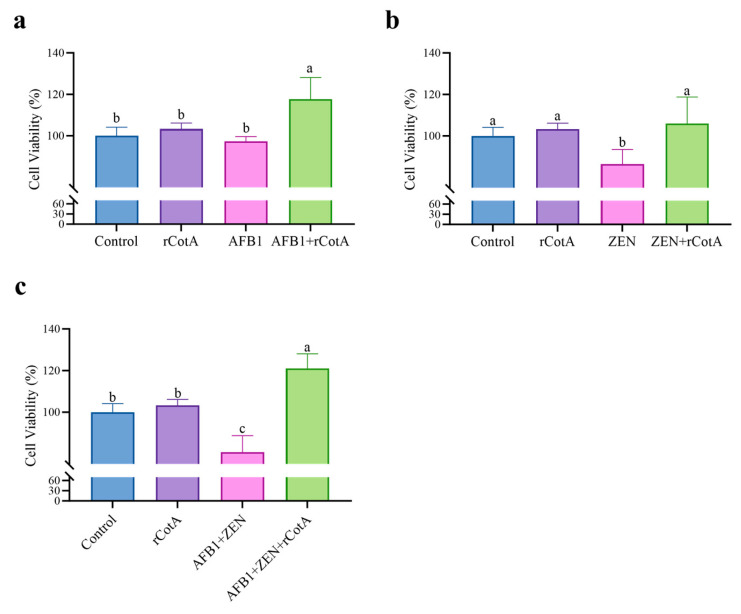
Detoxification effect of rCotA on AFB1 and/or ZEN in Raw 264.7 cells. (**a**) Detoxification effect of rCotA on AFB1 compared to the PBS-treated group. (**b**) Detoxification effect of rCotA on ZEN compared to the PBS-treated group. (**c**) Detoxification effect of rCotA on “AFB1 + ZEN” compared to the PBS-treated group. The results are presented as the mean ± SD of three independent experiments. Different lower-case letters indicate a significant difference between the two groups (*p* < 0.05).

The detoxification potential of rCotA was evaluated using the Raw264.7 cell. As shown in Figure 4a, exposure to 5 μg/mL AFB1, 20 μg/mL ZEN, or a combination of 5 μg/mL AFB1 and 20 μg/mL ZEN resulted in cell survival rates of 97.37%, 86.49%, and 83.84%, respectively, indicating that both AFB1 and ZEN were cytotoxic to Raw264.7 cells. In contrast, treatment with 30 μg/mL rCotA resulted in 100% cell viability for RAW 264.7 cells, indicating the non-toxic nature to eukaryotic cells. Furthermore, after incubation with rCotA (30 μg/mL), the cell viability in the presence of AFB1 and ZEN increased significantly to 117.74% and 105.58%, respectively, indicating that the toxicity of the degradation products is significantly reduced compared to the AFB1 and ZEN prototypes. These results support the non-toxic profile of rCotA and its potential as a safe and effective detoxification agent. 

### 2.5. Characteristic of rCotA Immobilized Chitosan Microspheres and Their Application to Contaminated Maize Meal

**Figure 5:**
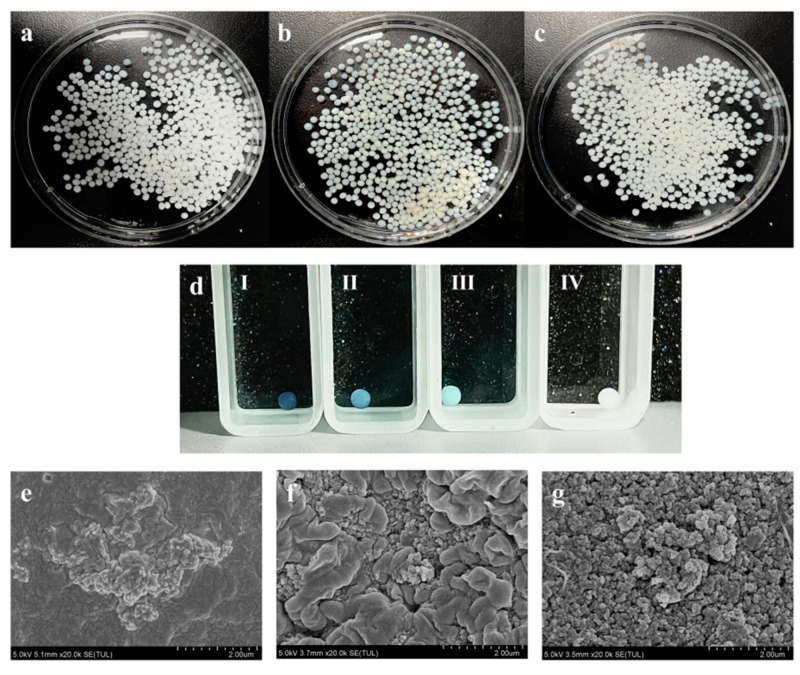
Morphology and characteristics of chitosan microspheres: (**a**) chitosan microspheres before activation; (**b**) chitosan microspheres cross-linked with glutaraldehyde; (**c**) chitosan microspheres immobilized with rCotA; (**d**) ABTS oxidation by chitosan microspheres with varying concentrations of immobilized rCotA: 2 mg/g (**I**), 1 mg/g (**II**), 0.5 mg/g (**III**), and 0 mg/g (**IV**); (**e**) scanning electron microscopy image of untreated chitosan microspheres; (**f**) scanning electron microscopy image of chitosan microspheres activated by glutaraldehyde; (**g**) scanning electron microscopy image of glutaraldehyde-activated chitosan microspheres immobilized with rCotA.

To enhance the reusability of rCotA, it was immobilized using chitosan microspheres. As shown in Figure 5a, the chitosan microspheres were homogeneous, creamy white spheres with a diameter of 2 to 3 mm, with regular morphology and good mechanical stability. After activation by glutaraldehyde, the microspheres maintained their morphology and size, though their color changed from milky white to pale yellow (Figure 5b). Immobilization with rCotA further deepened the color of the microspheres (Figure 5c), indicating successful enzyme binding. We further assessed the activity of immobilized rCotA by ABTS, and the result showed that chitosan microspheres immobilized with CotA laccase were able to oxidize the ABTS to ABTS^•+^, resulting in a green coloration on the surface of the microspheres, and the depth of the coloration deepened with increasing concentration of immobilized rCotA (Figure 5d-I,II,III). In contrast, glutaraldehyde-activated chitosan microspheres without rCotA failed to catalyze the oxidation of ABTS, and their surface color remained unchanged (Figure 5d-IV).

Scanning electron microscopy (SEM) analysis revealed that the surface of untreated chitosan microspheres was flat, smooth, and devoid of any pore structures, with small protrusions present (Figure 5e). Upon activation by glutaraldehyde, numerous protrusions and dense pores appeared on the surface (Figure 5f). After enzyme immobilization, the surface retained its protrusions, with rCotA clusters visible within the pores (Figure 5g). These findings confirm the successful immobilization of rCotA laccase within the glutaraldehyde-activated chitosan microspheres.

**Figure 6:**
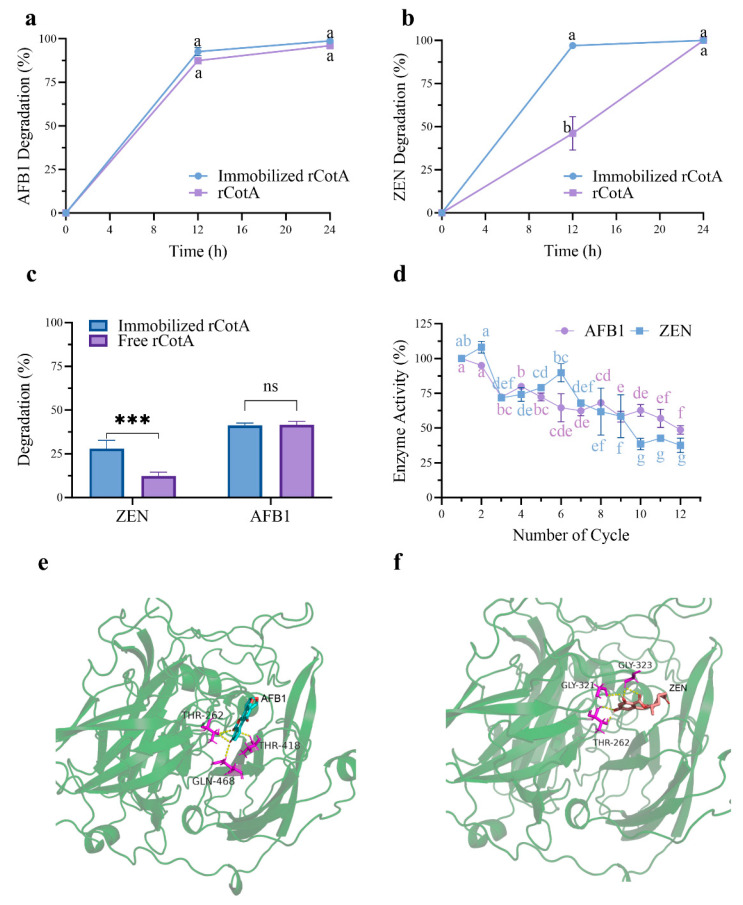
Degradation of AFB1 and ZEN by free and immobilized rCotA and reusability of the immobilized enzyme: (**a**) Degradation of AFB1 by free and immobilized rCotA. (**b**) Degradation of ZEN by free and immobilized rCotA. (**c**) Degradation of AFB1 and ZEN in contaminated maize meal by free and immobilized rCotA. The “ns” indicates no significant difference between the two groups, and asterisks indicate a significant difference between the two groups (*** *p* < 0.001). (**d**) Remaining activity of immobilized rCotA for AFB1 and ZEN degradation over multiple cycles. (**e**) The 3D binding pocket model of rCotA with AFB1. (**f**) The 3D binding pocket model of rCotA with ZEN. The results are given as the mean ± SD of three independent experiments. Different lower-case letters indicate a significant difference between the two groups (*p* < 0.05).

Finally, we successfully achieved chitosan-immobilized rCotA microspheres. Compared to free rCotA, the AFB1 and ZEN degradation rate increased from 87% to 92% and from 45% to 96%, respectively, at 12 h (Figure 6a,b). This result suggested that the rCotA- immobilized chitosan beads exhibited higher ZEN and AFB1 degradation activity. Additionally, in practical applications, we evaluated the efficacy of rCotA in degrading mycotoxins in contaminated maize meal. Chitosan-immobilized rCotA demonstrated better ZEN degradation (27.95%) compared to free rCotA (12.36%) under identical conditions. However, there was no significant difference in AFB1 degradation between the immobilized and free forms of rCotA (immobilized rCotA: 41.37%, free rCotA: 41.65%) (Figure 6c).

Furthermore, we assessed the stability and reusability of rCotA-immobilized chitosan microspheres over multiple usage cycles. The degradation activity of rCotA against AFB1 and ZEN decreased progressively with each cycle. After 12 cycles, immobilized rCotA retained 48.67% and 37.47% of its initial activity for AFB1 and ZEN degradation, respectively (Figure 6d). This suggests that chitosan-immobilized rCotA has potential for cost-effective reuse in industrial applications.

Molecular docking simulations were performed to elucidate the interactions between recombinant CotA (rCotA) and AFB1/ZEN. The analysis revealed distinct binding characteristics for each substrate. AFB1 was found to form four hydrogen bonds with the active site residues THR262, GLY321, and GLY323 of rCotA (Figure 6e). In contrast, ZEN established five hydrogen bonds with the enzyme, involving the residues THR262, THR418, and GLN468 (Figure 6f). These findings highlight the differing binding dynamics of AFB1 and ZEN, which may be pivotal in the enzyme’s ability to degrade both substrates simultaneously.

## 3. Discussion

Laccase, a copper-containing polyphenol oxidases, catalyzes the four-electron reduction of O_2_ to H_2_O coupled with the oxidation of phenolic compounds, which has been widely applied in many fields, such as the detoxification of mycotoxins and decolorization of industrial dyes. In the present study, recombinant laccase rCotA from *B. subtilis* ZJ-2019-1 was successfully expressed in *E. coli* as a soluble form and demonstrated efficient degradation of AFB1 and ZEN (Figure 1f and Figure 2). Previous research suggested that Cu^2+^ can promote laccase production in *Trametes velutina* 5930 [34]. This provides ideas for improving the yield of recombinant laccase in genetic engineering. Similar results were found in our study where 0.5 mM Cu^2+^ significantly promoted the expression level of rCotA in *E. coli* (Figure 1e). Although *E. coli* was selected due to its rapid growth and straightforward genetic manipulation, we recognize its limitations in protein folding and post-translational modifications for complex enzymes like laccases. Future work may investigate alternative systems, such as yeast (*Pichia pastoris* or *Saccharomyces cerevisiae*), to further optimize the production and functionality of laccase enzymes. The degradation activity of rCotA toward AFB1 and ZEN was most significant at pH 7.0–8.0, but it decreased dramatically to 13.53%–59.44% at pH 6.0 and only 0.00%–20.52% at pH 5.0, indicating reduced stability of rCotA under acidic conditions (Figure 2a,b). This observation is similar to a previous report indicating that BsCotA showed higher activity at pH above 7.0 [26]. Laccase is known for its resistance to high temperatures. For example, Wang et al. reported that the optimal temperature for BsCotA was 60 °C [26]. In our study, the AFB1/ZEN degradation capacity of rCotA increased with rising temperature, and reached a maximum at 70 °C, though it declined at 80 °C (Figure 2c,d). Similarly, another laccase identified from *Bacillus licheniformis* ANSB821 also exhibited maximum activity at 70 °C [25]. These results provide the possibility of high temperature processing for rCotA applications.

The use of mediator systems in laccase-catalyzed mycotoxins degradation has been extensively reported [35]. For instance, Ery4 laccase alone could not directly oxidize AFB1 and ZEN, but mediators like syringaldehyde significantly enhanced its catalytic efficiency, achieving 86% degradation of AFB1 and 100% for ZEN [27]. Similarly, BsCotA laccase from *Bacillus subtilis*, demonstrated only 1.7% and 1.6% degradation rates for AFB1 and ZEN, respectively, in a 10 h reaction at 30 °C [35]. However, the addition of methyl syringate markedly improved BsCotA’s degradation performance, with 94.2% degradation of AFB1 and 100% degradation of ZEN [35]. In contrast, our study found that rCotA exhibited up to 91.99% degradation of AFB1 and 98.34% of ZEN after 24 h of incubation without any mediator (Figure 2), suggesting that rCotA can efficiently degrade AFB1 and ZEN without relying on the mediator. One reason for the observed discrepancy in degradation efficiency may be due to the different temperature and incubation time of the reaction systems, i.e., the optimal reaction temperature (70 °C) for 24 h in our study (Figure 2) and 30 °C for 10 h in the previous investigation. When we reduced the incubation time to 2 h, the degradation of AFB1 and ZEN by rCotA was only 13.73% and 4.3%, respectively. Under shorter incubation conditions (1 h), mediators like acetosyringone and syringaldehyde significantly enhanced rCotA’s mycotoxin degradation ability. This observation aligns with previous findings, where syringaldehyde and acetosyringone were also the most effective mediators for Ery4 laccase and BsCotA in AFB1 and ZEN degradation [27]. Compared to alkaline mediators (matrine and sophoridin), acetosyringone and syringaldehyde, as phenolic compounds containing syringyl subunits, demonstrate stronger AFB1 and ZEN degradation capabilities. This enhanced performance may be attributed to their methoxy groups, which facilitate electron transfer during the laccase-catalyzed oxidation reactions [35,36].

While the laccase mediator system shows excellent potential in assisting laccase with toxin degradation in vitro, further research is needed to determine its efficacy in vivo. For example, Wang et al. tested a laccase mediator system in a hydra model to evaluate AFB1 detoxification. Hydra exposed to AFB1 alone collapsed after 18 h, while those exposed to AFB1 pre-treated with the laccase mediator system remained alive [35]. In addition, ingestion of mycotoxin-contaminated feed is a serious health hazard for livestock and poultry. When laccase and mediator are fed together to animals, the question arises as to whether there happens to be an effective involvement of mediator molecules when laccase interacts with toxins in the gastrointestinal environment of animals. This also requires further study in the future.

Enzyme immobilization technology, entailing the attachment of enzymes to a support matrix to form immobilized enzyme catalysts, encompasses methods such as carrier or matrix binding, encapsulation or entrapment, and cross-linking. Chitosan can be easily converted into microspheres in copolymer form, a well-established immobilization method that is advantageous for its safety, non-toxicity, and cost-effectiveness [37]. The principle of chitosan immobilization involves the reaction of one aldehyde group (-CHO) of glutaraldehyde with the amine group (-NH_2_) of chitosan, thus covalently linking the other aldehyde group with the enzyme [38,39]. In our study, the degradation activity of ZEN by chitosan microsphere-immobilized rCotA was enhanced by 110.37% when AFB1 was increased by only 5.8% compared to free rCotA (Figure 6b), which may be attributed to the improved thermal stability of laccase by immobilization [40,41]. Maize and its by-products are highly contaminated with mycotoxins, posing a serious threat to livestock and human health. In this study, we explored the effectiveness of free and immobilized rCotA in degrading AFB1 and ZEN in corn meal collected from Hebei Province, China, which was naturally contaminated with 80 μg/kg AFB1 and 3 mg/kg ZEN. Both free and immobilized rCotA exhibited 41% ZEN detoxification in naturally contaminated corn meal, while immobilized rCotA degraded 25% of AFB1, compared to free rCotA (15%) (Figure 6c). This could be due to the immobilized material providing protection and improved stability for rCotA. In a similar study by Guo et al., the ZEN removal rate in cornmeal by free and immobilized CotA laccase from *Bacillus licheniformis* reached 70% and 90%, respectively [25]. However, it is noteworthy that in our study, we applied free and immobilized rCotA directly to naturally moldy cornmeal, which better reflects real application environments, whereas Guo et al. used a ZEN standard spiked into the cornmeal, which may lead to a significant difference in degradation rates.

Reusability is an important advantage of immobilized enzymes over free enzymes. For example, Bo et al. developed a biomimetic core–shell structure, PDA@Lac, by immobilizing CotA laccase with polydopamine encapsulation, which retained approximately 75% of its relative activity after 10 cycles of reuse [32]. In our study, the immobilized rCotA laccase maintained significant degradation activity, with 48.67% for AFB1 and 37.47% for ZEN, after 12 reuse cycles (Figure 6d). This suggests that immobilized CotA has moderate recovery capacity over multiple cycles. However, we observed a gradual decline in ZEN degradation by immobilized CotA laccase with increasing reuse cycles. This reduction may be attributed to the partial inactivation or leakage of enzyme molecules during each cycle of operation. Despite this, the data indicate that immobilized CotA laccase has greater potential as a commercial biocatalyst for ZEN detoxification in cornmeal compared to free rCotA. In industrial settings, mediator-assisted or immobilization rCotA can be integrated into processing lines to enhance the detoxification of contaminated food and feed. Variations in temperature, pH, and the presence of substrates or inhibitors can impact enzyme activity. For instance, higher temperatures and lower pH may denature rCotA. To further enhance the efficiency and stability of immobilized enzymes, future studies should focus on optimizing immobilization methods (i.e., nanoparticle or layer-by-layer) and materials (i.e., silica or alginate beads). Improvements in these areas could lead to more effective and durable biocatalysts for large-scale applications in mycotoxin detoxification.

## 4. Conclusions

Our study highlights the effectiveness of natural redox mediators in enhancing the degradation efficiency of rCotA and demonstrates the advantages of rCotA immobilization on chitosan, resulting in significantly improved degradation rates in contaminated maize meal. Firstly, laccase CotA from *Bacillus subtilis* ZJ-2019-1 was successively expressed in *E. coli*, with an activity of 247.2 U/L. Free rCotA showed the most efficient degradation of AFB1 (93.18%) and ZEN (98.5%) at pH 7.0 and 70 °C. The presence of redox mediators including acetosyringone and syringaldehyde could enhance AFB1 and ZEN degradation by rCotA. The immobilization of rCotA laccase onto chitosan microspheres improved its catalytic efficiency towards AFB1 and ZEN. Immobilized rCotA laccase could achieve 25% degradation of AFB1 and 41% degradation of ZEN in corn meal. Additionally, the immobilized enzyme maintained substantial activity over 12 reuse cycles, with 48.67% AFB1 and 37.47% ZEN degradation. Practically, this mediator-assisted or immobilization rCotA could be incorporated into feed and food processing systems to mitigate the risks associated with mycotoxin contamination.

## 5. Materials and Methods

### 5.1. Chemicals, Reagents, and Microbial Strains

Aflatoxin B1, zearalenone standards (≥99.0%) were purchased from Pribolab (Qingdao, China). 2, 2′-biazobis (3-ethylbenzothiazoline-6-sulfonate) (ABTS) (≥98.0%), isopropyl-β-d-thiogalactopyranoside (IPTG) (≥99%), acetyl butyrophenone (≥97%), and syringaldehyde (≥99.8%) were obtained from Sigma-Aldrich (St. Louis, MO, USA). The expression host cell *E. coli* BL2 (DE3) and the expression vector pEASY-Blunt E1 were purchased from TransGen Biotech (Beijing, China). Other reagents such as ampicillin (≥90.0%), vanillin (≥99.7%), matrine (≥98%), sophoridin (≥98%), chitosan, methanol (≥99.8%), and acetonitrile (≥99.8%) were purchased from Sinopharm Chemical Reagent Co., Ltd. (Shanghai, China). Cell Counting Kit-8 was obtained from Byeotime (Beijing China). Coomassie Brilliant Blue was purchased from Solarbio (Beijing China). Corn protein powder was preserved in our laboratory. SDS-PAGE assay kit was purchased from Bio-Rad (Beijing China)

### 5.2. Determination of AFB1 and ZEN by High-Performance Liquid Chromatography (HPLC)

To measure the degradation rates of AFB1 and ZEN by rCotA, a 5 ppm solution of either AFB1 or ZEN was prepared in PBS buffer, and 20 µg/mL of rCotA was added. At various time points, 200 μL of the AFB1/ZNE-containing samples were taken and placed into a 1.5 mL sterile centrifuge tube. We added 600 μL of methanol and mixed it using a vortex mixer for 1 min. Then, it was centrifuged at 10,625× *g* for 10 min at room temperature. The supernatant was collected and filtered through a 0.22 μm filtration membrane, and then transferred into an HPLC (Shimadzu LC-20, Kyoto, Japan) vial for subsequent analysis.

Detection conditions for AFB1 by HPLC: The column used was Agilent SB-C18 (150 mm × 4.6 mm, 5 μm). The mobile phase consisted of methanol/acetonitrile/water in a ratio of 22:22:56. The column temperature was 40°C, with an injection volume of 10 μL and a flow rate of 1.0 mL/min. The fluorescence detector was used with an excitation wavelength (Ex) of 365 nm and an emission wavelength (Em) of 430 nm. For ZEN detection by HPLC, the same column (Agilent SB-C18, 150 mm × 4.6 mm, 5 μm) was used. The mobile phase was methanol/acetonitrile/water in a ratio of 8:46:46. The column temperature was set at 30°C, with an injection volume of 10 μL and a flow rate of 1.0 mL/min. The fluorescence detector was set to an excitation wavelength (Ex) of 235 nm and an emission wavelength (Em) of 460 nm. The limit of detection (LOD) was 3 µg/kg, and the limit of quantification (LOQ) was 10 μg/kg. 

### 5.3. Cloning, Expression, and Purification of rCotA

Genomic DNA of *Bacillus subtilis* ZJ-2019-1 was extracted using the TIANamp Bacteria DNA Kit (TIANGEN, China) as the template. The CotA gene was amplified by PCR using the forward primer 5′-ATGACACTTGAAAAATTTGTGGATGC-3′ and reverse primer 5′-TTATTTATGGGGATCTGTTATATC-3′. The purified PCR product was subsequently inserted into the pEASY-Blunt E1 vector. The recombinant plasmid, pEASY-Blunt E1-CotA, was transformed into Escherichia coli BL21 (DE3) for the expression of CotA with an N-terminal His-tag.

The *E. coli* cells carrying the pEASY-Blunt E1-CotA vector were grown in LB medium supplemented with 50 μg/mL ampicillin. When the culture reached an OD_600_ of 0.6–0.8, it was transferred to fresh LB medium containing varying concentrations of CuSO_4_ (0, 0.1, 0.5, 1 mM) and IPTG (0.05, 0.1, 0.2, 0.3 mg/mL). The cultures were incubated for 18 h at 16 °C with shaking (150 rpm). After incubation, the cells were harvested by centrifugation (8000× *g*, 25 min, 4 °C), and the pellet was resuspended in lysis buffer (25 mM Tris, 500 mM NaCl, pH 6.9).

The cells were disrupted by sonication on ice, and the cell debris was removed by centrifugation (10,000× *g*, 20 min, 4 °C). The recombinant CotA laccase, containing an N-terminal 6 × His-tag, was purified using a Ni^2+^-NTA column (Cytivia HisTrapTMHP, Amershanm, UK). The concentration of purified rCotA was determined using the Bradford Protein Assay Kit (TIANGEN, Beijing, China). The laccase activity of rCotA was confirmed by the oxidation of the classical laccase substrate ABTS, with the reaction product showing a maximum absorption at 420 nm (ε420 = 3.6 × 10^4^ M^−1^ cm^−1^). Enzyme activity was quantified as the amount of enzyme that converts 1 micromole of ABTS in one minute under specific conditions. The reaction was conducted at different pH levels (2, 3, 4, 5, 6, 7, 8, and 10) at 25 °C. Assay reproducibility was ensured through triplicate measurements, confirming the reliability of the enzymatic activity results.

### 5.4. Enzymatic Properties as Well as Kinetics of ZEN as Well as AFB1 Degradation by rCotA

To evaluate the effects of temperature and pH on the activity of rCotA, a degradation test was performed in a 500 μL reaction system under varying pH levels (4, 5, 6, 7, and 8) and temperatures (50, 60, 70, and 80 °C). The reaction system contained 2 μg/mL of AFB1 or ZEN and 20 μg/mL of rCotA laccase. At time points 0, 4, 8, 12, 16, 20, and 24 h, the reaction was terminated by adding methanol at three times the volume of the reaction mixture. The contents of AFB1 and ZEN in the system were then measured using HPLC, and their respective degradation rates were calculated based on the chromatographic changes in peak area.

To assess the effect of various ions on the activity of rCotA laccase, the ion concentration in the reaction system was maintained at 5 mmol/L, with AFB1 or ZEN at a concentration of 2 μg/mL, and rCotA at a final concentration of 20 μg/mL. The reaction was conducted at 60 °C and pH 7.0. Samples were taken at 0 and 24 h, and the reaction was terminated by adding methanol at three times the volume. The residual AFB1 or ZEN content in the reaction mixture was measured, and the degradation rate was calculated.

### 5.5. Degradation of AFB1 and ZEN by the rCotA Mediator System

Five natural mediators (acetosyringone, syringaldehyde, vanillin, matrine, and sophoridin) were selected to evaluate their potential in assisting the degradation of AFB1 or ZEN by rCotA. Briefly, the rCotA laccase mediator system was tested with a concentration of 10 μg/mL for each toxin. The final concentration of rCotA was set at 20 μg/mL, and the mediator concentration was 1 mM. The reaction was terminated by adding methanol at three times the volume of the reaction mixture at 1 and 2 h, respectively. The degradation rates of AFB1 and ZEN were then determined by the HPLC method, as described in Section 5.2.

### 5.6. Cytotoxicity Assay of rCotA Degradation Products

The cytotoxicity of AFB1, ZEN, and their degradation products by rCotA was assessed using Cell Counting Kit-8 (CCK-8). Mouse macrophage RAW264.7 cells (2.5 × 10^4^ cells/mL, 100 μL) were seeded into a 96-well plate and incubated at 37 °C with 5% CO_2_ (*v*/*v*) for 24 h. The concentrations of mycotoxins were 5 μg/mL for AFB1, 20 μg/mL for ZEN, and 30 μg/mL for rCotA.

The experimental groups included a PBS blank control group, an rCotA group, a mycotoxin group, and a group with mycotoxins degraded by rCotA for 24 h. The cells were treated with these solutions accordingly. After treatment, 10 μL of CCK-8 solution was added to each well. Following a 1 h incubation, the absorbance (OD) was measured at 450 nm using a microplate reader.

### 5.7. Immobilized rCotA Preparation and Attenuation of Mycotoxin Degradation Rate

We weighed 0.5 g of chitosan and dissolved it in 20 mL of 1% glacial acetic acid (*v*/*v*), stirring until fully dissolved. Using a 1 mL syringe, this solution was slowly added drop by drop into 2 mol/L NaOH to form chitosan microspheres with a smooth surface. After formation, the NaOH was washed away from the surface of the microspheres. Then, they were immersed in 8% glutaraldehyde solution (*v*/*v*) for overnight cross-linking. They were washed again to remove residual glutaraldehyde from the surface. To immobilize rCotA onto the chitosan microspheres, the ratio of chitosan to rCotA should be 1 g chitosan to 0.5, 1, or 2 mg rCotA, and the immobilization should be performed at room temperature for 4–6 h. For mycotoxin degradation studies, we used the chitosan microspheres immobilized with rCotA in a reaction system of 1 mL sodium phosphate buffer (10 mM, pH 7.0), with a final concentration of 20 μg rCotA. AFB1 or ZEN content was 5 μg/mL, the reaction temperature was 60 °C, and the percentage of mycotoxin degradation activity was calculated separately for different number of cycles. 

To prepare samples for scanning electron microscope (SEM) analysis, the chitosan microspheres, glutaraldehyde cross-link chitosan microspheres, and chitosan microspheres immobilized with rCotA were subjected to a gradient dehydration process using 50%, 70%, 100%, and 100% ethanol (*v*/*v*), with each concentration applied for 10 min. After dehydration, the samples were transferred from 100% ethanol to a supercritical dryer for critical point drying, which typically takes about 1 h. Once dried, the samples from the critical point dryer were fixed onto the sample stage using conductive tape, and coated with gold. Finally, we observed the overall morphology and surface condition of the samples using a Quanta 250 scanning electron microscope.

### 5.8. Degradation of Mycotoxins in Naturally Contaminated Corn Protein Powder 

Corn protein powder naturally contaminated with 80 μg/kg of AFB1 and 3 mg/kg of ZEN was used to evaluate the degradation efficiency of immobilized and free rCotA. The contaminated powder was ground and 10 g of each sample was weighed and suspended in 40 mL of solution containing either immobilized or free rCotA, with a final enzyme concentration of 0.75 μg/mL. Controls were performed using chitosan microspheres without immobilized rCotA and PBS. All samples were incubated at 60 °C for 24 h. After incubation, the supernatant was removed by centrifugation, and 0.5 g of sodium chloride and 25 mL of an extraction solution (acetonitrile/water, 4:1, *v*/*v*) were added to the remaining solid phase. The mixture was subjected to agitation at shaker (200 rpm) for 60 min, followed by centrifugation. The resulting supernatant was filtered through a 0.22 μm membrane and analyzed using HPLC to determine the degradation rates of AFB1 and ZEN.

### 5.9. Interaction between rCotA and AFB1/ZEN 

The binding sites of AFB1 and ZEN to rCotA were determined through molecular docking using AutoDock Vina. The structure of rCotA was modeled using AlphaFold2. The 3D structures of AFB1 (Compound CID: 186907) and ZEN (Compound CID: 5281576) were obtained from PubChem as ligands. Based on previous reports regarding the active sites of CotA [42], a grid box of 40 × 40 × 40 points was defined with a center at coordinates (x = 2.565, y = −0.584, z = −12.655) and a grid spacing of 0.375 Å. All molecules were kept rigid during the docking process, and the final complex conformation with the minimum binding energy was selected as the putative binding site. The results were visualized using PyMOL 2.3.

### 5.10. Statistical Analysis

The test data were analyzed using one-way ANOVA for comparing multiple groups and *t*-test for pairwise comparisons with GraphPad Prism version 8 software (GraphPad Software, San Diego, CA, USA). Prior to ANOVA, we assessed data normality and homoscedasticity to ensure the assumptions of the test were met. The significance of differences was assessed using Duncan’s multiple comparisons method, with *p* < 0.05 as significance, using a 95% confidence level. Results are expressed as mean ± standard deviation (*n* = 3).

## Data Availability

The data presented in this study are available on request from the corresponding authors.
